# Supplementary material for: Assessment of drug-susceptible and multidrug-resistant tuberculosis (MDR-TB) in the Central Region of Somalia: A 3-year retrospective study
Source: PLOS Glob Public Health. 2023 Sep 7;3(9):e0002319. doi: 10.1371/journal.pgph.0002319 (PMC10484424; doi:10.1371/journal.pgph.0002319)
Supplement: S1 File — (PDF) [file pgph.0002319.s001.pdf]

Date: 22/02/2022
Ref.No. EAU: 10984

Subject: Research Approval Letter

Dear Researcher

The institutional Review Board Committee in its meeting held in 6 February, 2022 has reviewed and discussed your application to conduct the research proposal entitled "Retrospective study of multi-drug resistance TB".

It was understood that this study was planned according to scientific and ethical rules.

Everyone agreed that the committee should be informed of any new information on the study whose topic of study was discussed and its results. Any alteration to the project must be notified to the Committee.

IRB COMMITTEE MEMBERS	Title	Signature
Saed Mohamed Hassan	Chair	
Mohamed Saed Mohamed	Member	
Mohamed Abdullahi Farah	Member	
Abdirizak Abdulkadir Yussuf	Member	

IRB decision: Approved

Saed Mohamed Hassan

Acedemic director, EAU Galkacyo campus
