# Supplementary material for: Assessment of drug-susceptible and multidrug-resistant tuberculosis (MDR-TB) in the Central Region of Somalia: A 3-year retrospective study
Source: PLOS Glob Public Health. 2023 Sep 7;3(9):e0002319. doi: 10.1371/journal.pgph.0002319 (PMC10484424; doi:10.1371/journal.pgph.0002319)
Supplement: Attachment — Submitted filename: Response to Reviewer_PGPH-D-23-00423.docx [file pgph.0002319.s003.docx]

**Author responses to Reviewer’s Comments: PGPH-D-23-00423**
*Assessment of drug-susceptible and multidrug-resistant tuberculosis (MDR-TB) in the Central Region of Somalia: A 3-year Retrospective Study*
PLOS Global Public Health

Dear Editor:

Thank you for your review of this manuscript and please find my responses to your request for a revision below. Please note my reference to line numbering corresponds to that in the *marked-up version* where changes are being tracked by MS Word. Due to the addition and deletion of text in the marked-up version, the line numbering may be different than that in the originally submitted draft of the manuscript. For each reviewer/editor comment, I’ve indicated the action taken, if any, and the corresponding location in the manuscript.

Sincerely,

Shu-Hua Wang, MD	Rafael Vieira, PhD	Aamir Muse Osman

[Wang.1055@osu.edu](mailto:Wang.1055@osu.edu)	[rvieira@uncc.edu](mailto:rvieira@uncc.edu)	[aamirmuse@gmail.com](mailto:aamirmuse@gmail.com)

Corresponding authors

**General Comments:**

Journal Requirements:

1. Please send a completed 'Competing Interests' statement, including any COIs declared by your co-authors. If you have no competing interests to declare, please state "The authors have declared that no competing interests exist". Otherwise please declare all competing interests beginning with the statement "I have read the journal's policy and the authors of this manuscript have the following competing interests:"

*Authors response: The authors have declared that no competing interests exist. (line 248)*

2. Please provide separate figure files in .tif or .eps format only and remove any figures embedded in your manuscript file. Please also ensure all files are under our size limit of 10MB.

For more information about figure files please see our guidelines:

[https://journals.plos.org/globalpublichealth/s/figures](https://urldefense.com/v3/__https:/journals.plos.org/globalpublichealth/s/figures__;!!AU3bcTlGKuA!GTjQbjIz2Jn5v84uJVQlpAS6VUo2smBcE0Oo0yw8oPP5ZwUyVnA52t5FrvIIki6TvIlaWKbqongMwPou_9QN_Q$) 
[https://journals.plos.org/globalpublichealth/s/figures#loc-file-requirement](https://urldefense.com/v3/__https:/journals.plos.org/globalpublichealth/s/figures*loc-file-requirement__;Iw!!AU3bcTlGKuA!GTjQbjIz2Jn5v84uJVQlpAS6VUo2smBcE0Oo0yw8oPP5ZwUyVnA52t5FrvIIki6TvIlaWKbqongMwPqNlpGiMg$)

*Authors’ response: We have removed the figures and have added separate files.*

*Figure 1 – page 5 (lines 107-132) removed*

3. We do not publish any copyright or trademark symbols that usually accompany proprietary names, eg  ©, ®, ™  (e.g. next to drug or reagent names). Please remove all instances of trademark/copyright symbols throughout the text, including ® on page 9.

*Authors’ response: We have removed copyright symbols (Page 4 – line 88)*

4. We have noticed that you have uploaded Supporting Information files, but you have not included a list of legends. Please add a full list of legends for your Supporting Information files after the references list.

*Authors’ response: We have listed supporting files after reference section (Page 14, line 304-306)*

Supporting files:

1. Institutional Review Board approval document
2. Figure 1. Flow chart for evaluation of patients with tuberculosis

*The list of legends have been updated after the reference list.*

**Reviewer #1:** The author tried to evaluate the prevalence of MDR-TB in the Central Region of Somalia based on a single-center 3-year laboratory records of GeneXpert MTB/RIF results. A total of 714 unique individuals was finally enrolled and among these, 95 were defined as MDR-TB. In general, this research article was rather small with quite featureless results. The collected baseline data just included gender, age and region. More factors associated with MDR-TB, such as history of prior TB, close contact with TB, other lung disease, co-morbidities (HIV infection, diabetes), tobacco or alcohol use and so on, should be taken into account. In addition, the laboratory results were limited to GeneXpert results only. Drug susceptibility testing to indicate drug resistance other than rifampin should also be carried out.

*Authors’ response: We acknowledge that the scope of our study was limited, and we agree that additional factors associated with MDR-TB should be considered to provide a more comprehensive analysis. Unfortunately, due to resource constraints and the retrospective nature of our study relying on laboratory records, we were unable to collect all the suggested data.*

*We understand the importance of considering these factors in assessing MDR-TB prevalence and the impact they may have on the interpretation of our results. In future studies, we plan to incorporate a more extensive range of variables to provide a more robust analysis. Regarding the limitation of using only GeneXpert results for drug susceptibility testing, we acknowledge the necessity of broader drug resistance testing beyond rifampin. Unfortunately, in Somalia, access to comprehensive drug susceptibility testing was limited. We recognize the importance of expanding the testing methods in future investigations to capture a more accurate representation of drug resistance profiles. We sincerely appreciate your feedback, and we will take your suggestions into consideration for future research endeavors. We hope that despite the limitations, our study provides a valuable contribution to the understanding of MDR-TB prevalence in the Central Region of Somalia.*

**Reviewer #2:** This is an interesting study but falls short in analysis. I suggest the following major revisions be done:
1. Plot trends in DS-TB separately from trends in MDR-TB from 2019 to 2022 and stratify them according to all the predictor variables: gender, age, year, and regions ( these are 8 plots, you could put them in a panel)

***Authors’ response:*** *Thank you for the suggestion. We were not able to perform the plot graphs. We hope that the suggested additional Table 2 would provide the information comparing factors associated with drug susceptible and drug resistant by gender, age, year (pre-covid and covid) and region (central vs non-central).*

2. Perform chi-squared tests as you did, but in each of the two classes (separate for DS-TB and separate for MDR-TB) - these will be two tables. Try using Fisher’s test in case the chi-square test fails.

***Authors’ response:*** *The updated analysis is completed in table 2 (page 8 line 189) and the findings summarized in result section (line 164 – 167)*

3. Issues with logistic regression: First remember you sampled hospital records and so the sample size divisions between those with DS-TB and MDR-TB were not pre-determined in a sample size collection. The sample size is unbalanced, and this must be the reason why you get non-significant results. This is because logistic regression fits a Maximum Likelihood Estimate by minimising an objective function which is evaluated at all the data points. If the data is unbalanced, then the minimisation will be unbalanced too. In your car, there are 95 1s (for MDR-TB+ ) and 619 0s (for DS or MDR-TB-). Another way is to take the 95 who are MDR-TB and find their ‘match’, for example, match by gender, age-groups, year and regions as much as possible until you get a more balanced sample (you could take for each MDR-TB + you take two DS-TB members) and repeat the analysis regression analyses

*Authors’ response: Thank you for the explanation. As per your suggestion below, we have performed the Odds Ratio.*

4. Or instead of logistic regressions, perform analysis of odds ratios/relative risks. You do this by creating a 2 by 2 table in which the outcome (DS-BT vs MDR-TB) is compared to a dichotomized explanatory variable (you could dichotomize age into young vs old; gender is already dichotomized, year could be pre-covid 19 vs in-covid 19, region could be ‘central Somalia vs non-central Somalia).

*Authors’ response: Odds Ratio is performed as suggested in Table 2.*

5. The number and type of predictor variables collected could just by the nature of problem not be the appropriate ones, and that is why you have negative results.

*Authors’ response: Thank you for your feedback and insight. We appreciate your perspective on the potential limitations of the predictor variables collected in our study.*

6. These suggested analyses will enrich your analysis and probably confirm whether your negative results are really because of an underlying population process and not due to shortcomings in statistical analysis.

*Authors’ response: Thank you for your valuable suggestions. We agree that the additional analyses you have proposed could provide further insights into our research and help us better understand.*
